# A CRISPR interference system for tunable gene expression integrated with a promoter library for *Eubacterium callanderi* KIST612, an acetogen of functional diversity and versatility

**DOI:** 10.1128/spectrum.03779-25

**Published:** 2026-05-18

**Authors:** Byeongchan Kang, Ji-Yeon Kim, Soyoung Oh, Jina Kweon, Hongseo Park, Minji Kim, In-Geol Choi, In Seop Chang

**Affiliations:** 1 Department of Environment and Energy Engineering, Gwangju Institute of Science and Technology 65419, Buk-gu, Gwangju, Republic of Korea; 2 Research Center for Innovative Energy and Carbon Optimized Synthesis for Chemicals (inn-ECOSysChem), Gwangju Institute of Science and Technology 65419, Buk-gu, Gwangju, Republic of Korea; 3 Department of Biotechnology, College of Life Sciences and Biotechnology, Korea University 34973, Seongbuk-gu, Seoul, Republic of Korea; University of Manitoba, Winnipeg, Canada

**Keywords:** synthetic promoter, promoter library, CRISPR interference, dead-Cas9, *Eubacterium callanderi* KIST612

## Abstract

Acetogens are key biocatalysts for carbon-neutral biorefineries, yet their metabolic engineering is limited by the lack of tunable transcriptional regulation systems. Here, we developed a synthetic promoter library for *Eubacterium callanderi* KIST612 and integrated it with a CRISPR interference (CRISPRi) system to establish precise and scalable gene regulation. Motif analysis of 3,109 putative native promoters revealed conserved and semi-conserved −35 and −10 elements, which were used to construct a promoter library spanning a >20-fold dynamic range of transcriptional strengths. The system was validated by knockdown of *pyrF*, where promoter strength directly determined repression efficiency (*R*^2^ = 0.92), with high-strength promoters achieving near-complete gene silencing. Application to lactate dehydrogenase (*ldh*) revealed that increasing promoter strength progressively reduced lactate production from 93.3% to 0.0% of control. This study establishes a versatile synthetic promoter-CRISPRi platform tailored for acetogens, enabling precise control of gene expression and mechanistic dissection of redox metabolism.

**IMPORTANCE:**

Transitioning to a carbon-neutral economy requires biocatalysts that can efficiently convert waste-derived substrates into valuable products. Acetogens are industrially relevant organisms for gas fermentation, but the lack of genetic toolkits tailored to their physiology has constrained metabolic engineering. We present the first synthetic promoter-CRISPRi platform specifically optimized for *Eubacterium callanderi* KIST612, a model acetogen with high industrial potential. This system provides tunable and predictable regulation of gene expression, extending from mild repression to a near-complete knockdown that could alternate gene deletion systems. This system could be used for not only advancing fundamental understanding of acetogen physiology but also providing a broadly applicable genetic toolbox for precision engineering of sustainable microbial biorefineries.

## INTRODUCTION

Concerns about climate change underscore the urgent need for a transition toward sustainable and carbon-neutral raw materials and energy sources. Current industrial society still relies heavily on fossil-derived energy and chemicals, creating substantial barriers to achieving carbon neutrality (1). Microbial biorefineries represent a promising solution, as they enable the carbon-neutral production of valuable resources by utilizing existing waste materials in accordance with the principles of the circular economy and environmental sustainability (2). In particular, the microbial conversion of C1 waste gases such as carbon monoxide (CO) and carbon dioxide (CO_2_) into value-added products has gained increasing attention as a strategy for large-scale carbon management (3–5).

**Fig 1:**
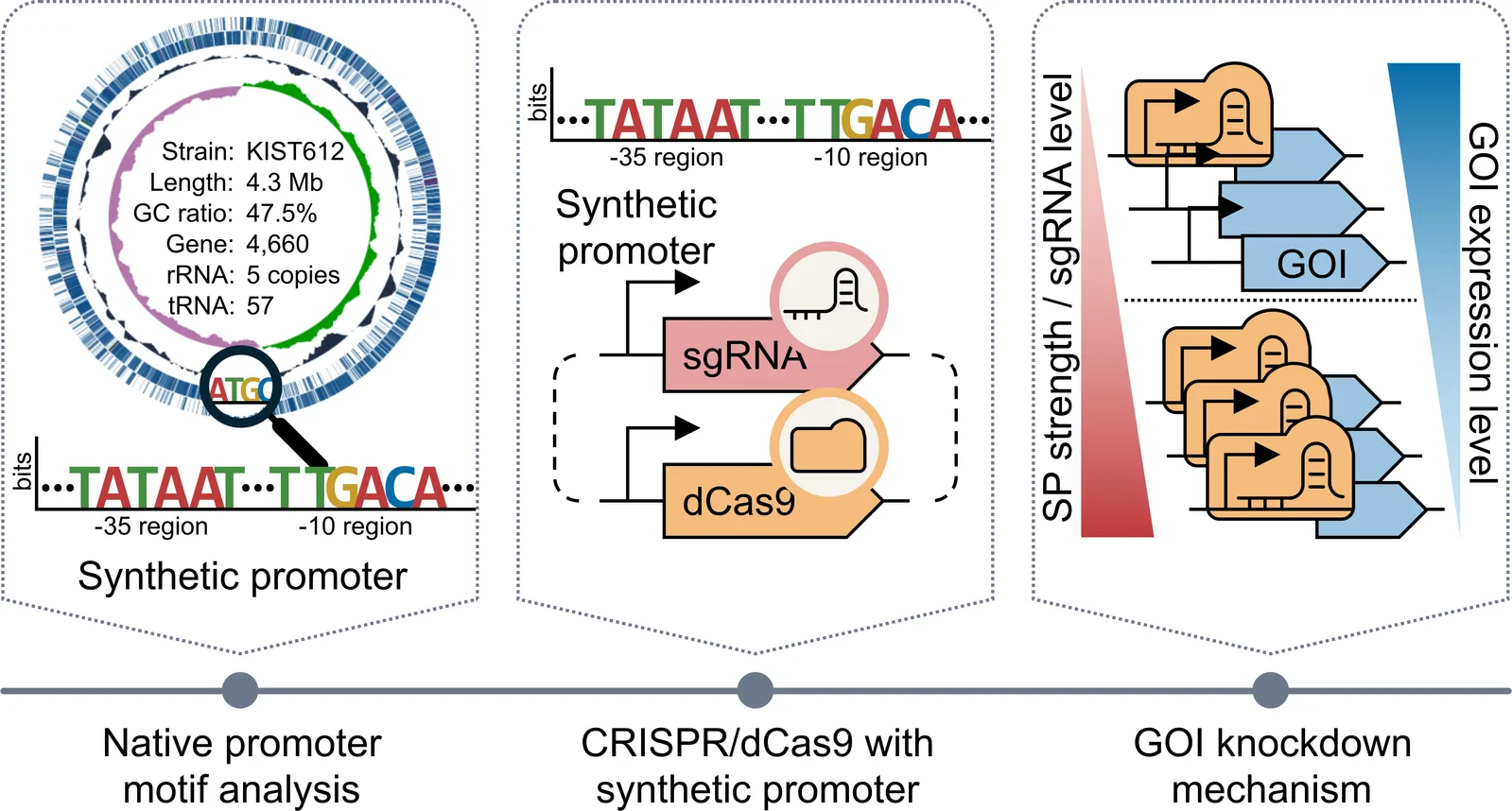
Scheme for construction of the CRISPRi-based platform with a synthetic promoter library. GOI, gene of interest; SP, synthetic promoter.

Acetogens play a pivotal role in both research and industrial applications aimed at converting waste, such as C1 gas, into valuable products (4). These microorganisms utilize the energy-efficient Wood-Ljungdahl pathway (also known as the reductive acetyl-CoA pathway) to fix C1 substrates into acetyl-CoA, thereby supporting the production of high-value metabolites and renewable energy resources (4, 6, 7). Most acetogens also possess the glycolytic Embden-Meyerhof pathway for sugar fermentation (4, 6, 8). The broad substrate utilization capacity of acetogens facilitates studies under both auto- and heterotrophic conditions. However, the construction of industrially competitive cell factories requires precise metabolic regulation, extending beyond simple gene overexpression or knockout to include fine-tuned transcriptional regulation (9, 10). Metabolic improvements that are not accompanied by fine-tuned regulation may disrupt intracellular homeostasis, such as redox balance in microbial catalysts, thereby complicating strain optimization. The regulation of gene expression is fundamental for metabolic engineering, with diverse methods available to influence transcription initiation and gene activity (11, 12). Among these strategies, promoters serve as the primary regulatory elements that directly control transcription initiation and thus play a central role in fine-tuning gene expression. Despite the importance of promoters, most available promoter libraries have been optimized for model industrial organisms, while acetogen-specific resources remain limited (13, 14) (Fig. 1). As a result, promoters derived from heterologous hosts often fail to provide predictable transcriptional strengths in acetogens without further validation. Developing promoter systems specifically adapted to acetogen strains, offering a tunable and predictable range of transcriptional activities, is therefore a critical step toward advancing acetogen engineering. To date, promoter systems have been reported in only a few acetogens, including *Acetobacterium woodii*, *Clostridium autoethanogenum*, and *Thermoanaerobacter kivui* (15–18).

Generally, promoter strength and transcription initiation levels in bacteria are influenced by the sequences, particularly in the −35 and −10 regions, along with adjacent nucleotides (19–21). By editing the sequences surrounding these core regions, it is possible to engineer synthetic promoters with a wide range of transcriptional strengths (9, 13). This approach allows for the generation of custom synthetic promoters to tailor the needs of the target organism (19). The “dead” Cas9-based CRISPR interference (CRISPRi) system was also developed for gene knockdown in *Eubacterium callanderi* KIST612 (22, 23). Finally, we integrated the two constructed systems for transcription level-controllable gene knockdown. The construction of a synthetic promoter library involving randomly designing promoter sequences and the CRISPRi system would enable the selection of promoters with distinct and optimized expression profiles, facilitating fine-tuned metabolic control in acetogens and supporting advanced research in microbial bioengineering and industrial bioprocesses (24).

In this study, we constructed a synthetic promoter library for *E. callanderi* KIST612 and integrated it with a CRISPRi system to enable precise, tunable regulation of gene expression (22, 23). *E. callanderi* KIST612 (formerly *Eubacterium limosum* KIST612) is a model acetogen with high CO tolerance and efficient gas-conversion rates (25, 26). Since its isolation in 1997, this strain has been extensively applied in microbial physiology studies and process development. Under optimized gas fermentation conditions, KIST612 can produce high titers of short-chain fatty acids (e.g., acetate, butyrate, and lactate). Especially, the reported butanol production in *Butyribacterium methylotrophicum*—which is now considered synonymous with KIST612—further enhances the biotechnological potential of this strain (27, 28). More recently, KIST612 has been engineered for ethanologenesis and other metabolic applications, further underscoring its industrial relevance (26, 29–31) (Table 1). Despite progress in developing genetic tools, the construction of strain-specific promoter systems that enable precise and tunable transcriptional regulation remains underexplored in acetogens. Promoter strength and transcription initiation are strongly influenced by sequence determinants, particularly within the −35 and −10 regions and their flanking nucleotides (20, 21, 32). By engineering synthetic promoters that vary in these motifs, it is possible to construct libraries spanning a broad range of transcriptional strengths. Such a promoter library would provide the foundation for fine-tuned metabolic regulation in acetogens, supporting both basic research and industrial bioprocesses.

**TABLE 1:** Research history and major studies of *E. callanderi* KIST612 (f. *E. limosum* KIST612)^*a*^

Year	Research objective and studies conducted	Research results highlighted	Ref.
1997	**Strain isolation and identification**	Strain isolation from anaerobic digester Strain identification as *E. limosum* KIST612 **Butyrate production** under the CO condition	(26)
1998	Growth characterization on CO fermentation	Physiological properties: growth rate (0.25 h^−1^), minimal CO partial pressure (60.8 kPa)	(33)
1999	**Defining minimal medium for CO fermentation**	Maximal **butyrate production (1.3 mM**) in a phosphate-buffered basal medium	(34)
2001	Optimization of a growth condition	Optimal CO partial pressure (281.5 kPa)	(35)
2007	Defining industrial medium and verification of high cell density fermentation	High cell densities by supplementing anaerobic digester fluid as a nitrogen source	(36)
2011	Genome sequencing and annotation	Completed whole-genome sequence (4.3 Mb, GenBank accession number: CP002273)	(32)
2014	Investigation of high CO mass transferred membrane bioreactor	High gas-liquid CO mass transfer system using hollow fiber membrane reactor	(37)
2015	Verification of electron bifurcative energy metabolism	ATP synthesis coupled with butyrate production pathway Na^+^-dependent energy conservation module	(38)
2017	Simulation and validation of CO mass transfer coefficient in bioreactor	Kinetic simulations validated experimental CO mass transfer coefficient (*k*_L_*a*, 13 h^-1^)	(39)
2017	**Finding an acetate (re)uptake phenomenon and acetate-assisted cell cultivation**	**Shift of main product to butyrate (5.7 mM**) under the CO condition by supplying acetate Enhancing cell growth (146%), growth rate (121%), and CO consumption (151%) by supplying acetate	(30)
2020	Development of genetic toolkits (transformation, constitutive expression system, CRISPR/Cas9)	Information on native promoters Native promoter-based constitutive overexpression system CRISPR/Cas9-based gene knockout system	(40)
2020	**Invention of “simultaneous gas feeding and cell-recycled bioreactor” and verification on CO condition**	Simultaneous gas feeding and cell-recycled reaction system Increased **biomass (9.7 g/L**) and **acetate (9.8 g/L**) concentration	(31)
2021	**Determination of CODH/ACS cluster and overexpression to redirect carbon flux**	Increased specific CO oxidation rate (3.1-fold) by CODH/ACS overexpression Increased **acetate production rate (1.4-fold**)	(41)
2021	Finding methanol as H_2_ metabolism facilitator	Methanol usability of the strain Enhancing H_2_/CO_2_ consumption by activating the Wood-Ljungdahl pathway using methanol Correlation between methanol and H_2_ metabolism	(42)
2022	Analysis type of essential minerals and concentrations and optimization for industrial medium	Reduced medium cost (84%) with enhanced acetate production (130%) by using sludge filtrate	(43)
2023	Determination of CO-sensing system	CO-sensing (0–30.39 kPa) whole-cell biosensor toolkit	(44)
2023	Reporting strategies for industrial application: acetogen-based syngas valorization	The way to produce high value-added chemicals	(4)
2023	Development of electrodialysis-integrated bioreactor as an economic product (acetate) separation system	Increased acetate productivity (20%) by applying recycled minerals Electrodialysis-based acetate separation (99.8% recovery efficiency)	(45)
2023	Strain reclassification	Reclassified *E. limosum* KIST612 as *E. callanderi* KIST612	(25)
2024	Upgrading electrodialysis-integrated bioreactor system using bipolar membrane electrodialysis	Demonstrated integrating gas fermentation with bipolar membrane electrodialysis (BPMED) BPMED acetate extraction rate (99.72%–99.82%)	(46)
2025	**Deciphering “acetate-reuptake” pathway and confirmation of “ethanologenesis” occurring via complete acetate uptake**	Determined and deciphered acetate reuptake (reductive acetogenesis) pathway Found the necessity of redox balance in metabolism Metabolic shift from acetogenesis to **ethanologenesis** occurs via complete acetate uptake	(29)

^*a*^ Studies reporting on acidogenesis/solventogenesis are shown in bold.

## RESULTS

### Determination of consensus sequence derived from native promoter regions

Generally, promoter sequences, located within the 5′ untranslated region (5′ UTR) of transcription units, contain consensus sigma factor recognition sites, including a −35 motif and a −10 motif (the Pribnow box). These consensus sequences can vary depending on the bacterial strain. Therefore, we analyzed the core promoter sequences containing these recognition sites in KIST612. Among the 4,660 putative genes, we identified 3,109 transcription units, and all corresponding 5′ UTRs were subjected to promoter motif analysis. The MEME-based search revealed three highly conserved sequence elements: a −35 motif (5′-TTGACA-3′), a −10 motif (5′-TATAAT-3′), and a ribosome-binding site (RBS, 5′-AGGAG-3′), all of which are identical to the consensus promoter sequences of *Escherichia coli* (47). In addition, we detected the transcriptional start site (+1, A), validating the reliability of motif identification. Within the predicted regions, we also identified two semi-conserved sequences corresponding to the −35 motif (5′-ATCATTGACAW-3′) and the −10 motif (5′-RTGNTATAATAAWADHA-3′), as well as a highly variable 12 bp spacer (N_12_) located between the two motifs. Based on this motif analysis, we designed synthetic promoters incorporating variations in the semi-conserved regions and their surrounding sequences. The transcriptional strengths of the partially randomized synthetic promoter library were then assessed by measuring green fluorescent protein (GFP) fluorescence intensity.

### Strength assessment of synthetic promoters based on expression of reporter genes

**Fig 2:**
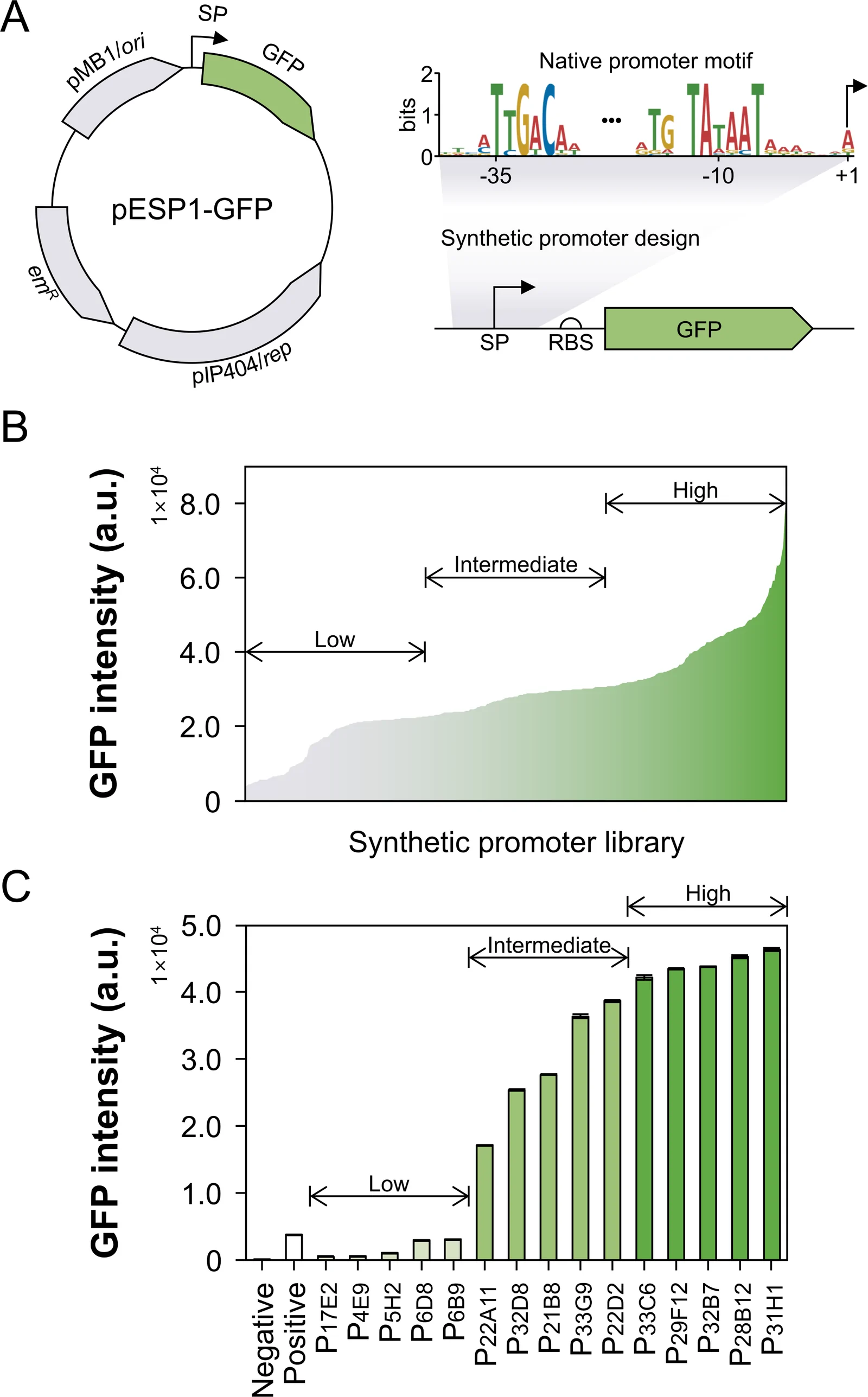
Construction and evaluation of synthetic promoter library in *E. coli* DH5α. (**A**) pESP1-GFP was constructed to confirm transcriptional efficiency of the synthetic promoter library. (**B**) Distribution of GFP intensities of 325 synthetic promoters. The promoters were categorized into three groups (low, intermediate, and high) based on the results. (**C**) Validation of GFP intensities for the selected synthetic promoters from each group. SP, synthetic promoter; GFP, green fluorescent protein; a.u., arbitrary unit; RBS, ribosome-binding site.

We constructed a synthetic promoter library using GFP as a reporter gene to enable rapid and effective classification of random promoters according to their strengths (Fig. 2A). Given that the predicted conserved promoter sequence was identical to that of *E. coli* and that our previous work has confirmed the functional compatibility of *E. callanderi* KIST612 promoters in *E. coli*, we used *E. coli* as the host strain for primary library screening (44, 47, 48). To ensure that reporter gene expression reflected promoter variability, the RBS (5′-ATTTGGACTATAGGAGGTCTTTATT-3′) from the 5′ UTR of ELI_3815 was exclusively used in all expression systems (40). The constructed plasmids were transformed into *E. coli* DH5α to quantify promoter-driven GFP expression. Given that 3,109 native promoter sequences were predicted, we evaluated GFP intensities from a total of 3,290 synthetic promoter transformants to ensure screening of a sufficient promoter set.

GFP fluorescence was measured using a microplate reader and normalized to OD_600_ values of each transformant (Fig. 2B). *E. coli* DH5α harboring pELMKO and pSB1C3::GFP were used as negative and positive controls, respectively (44). The negative control showed 3,643 arbitrary units (a.u.) of GFP intensity, whereas the positive control exhibited 114,006 a.u. The synthetic promoter library displayed a broad dynamic range of intensities, ranging from 3,864 to 84,568 a.u. Based on normalized GFP intensities, we selected three groups: the top 5% (high), middle 5% (intermediate), and bottom 5% (low) of transformants. We then sequenced the plasmids from these transformants and excluded candidates with mutations in the RBS or GFP sequence, yielding a total of 325 promoters. From these, 15 promoters (P_17E2_, P_4E9_, P_5H2_, P_6D8_, P_6B9_, P_22A11_, P_32D8_, P_21B8_, P_33G9_, P_22D2_, P_33C6_, P_29F12_, P_32B7_, P_28B12_, and P_31H1_) were selected for detailed characterization and evaluation (Table 2).

**TABLE 2:** Information of synthetic promoters used in the study

Group	Name	Sequences (5′→3′, 40 bp)
Low	P_17E2_	ATCATTGACAAAGCTCCCTACGGATGATATAATAATATAA
	P_4E9_	ATCATTGACAAGATAAGAAAACCGTGTTATAATAAAAACA
	P_5H2_	TCATTGACAAATAGGCGCATCGGTGATATAATAAAATTA
	P_6D8_	ATCATTGACATAAACCGTTAACAATGGTATAATAAAATAA
	P_6B9_	ATCATTGACAACTCACTTCGAGCGTGCTATAATAAAAACA
Intermediate	P_22A11_	ATCATTGACATGCGGGGACACCTATGATATAATAAAATAA
	P_32D8_	ATCATTGACAACTACCCGGCCTAATGGTATAATAATAATA
	P_21B8_	ATCATTGACATCATCTCCCCACAGTGCTATAATAATAATA
	P_33G9_	ATCATTGACAATGCAATCTCTACGTGCTATAATAAAAACA
	P_22D2_	ATCATTGACATTCCGGGCAGAGCATGATATAATAAAAATA
High	P_33C6_	ATCATTGACAAGGCAGCTTCTGTGTGATATAATAATAACA
	P_29F12_	ATCATTGACATCCAGCCGCGGCAGTGGTATAATAAAAACA
	P_32B7_	ATCATTGACATGTTGACTTGTACATGCTATAATAAAAGAA
	P_28B12_	ATCATTGACAATCAATTTCAGCGGTGATATAATAAAAACA
	P_31H1_	ATCATTGACATGCTTGTGTCTCAGTGCTATAATAATAACA

The intensities of the selected 15 promoters were reconfirmed by fluorescence spectroscopy (Fig. 2C). At 488/510 nm, the GFP intensities of the negative and positive controls were 142 ± 2 and 3,826 ± 34 a.u., respectively. The synthetic promoters exhibited a broad range of GFP intensities, spanning 3.8 ± 0.1- to 325.4 ± 6.1-fold relative to the negative control. Specifically, GFP intensities were as follows: P_17E2_, 544 ± 13; P_4E9_, 621 ± 29; P_5H2_, 1,058 ± 18; P_6D8_, 2,957 ± 31; P_6B9_, 3,103 ± 54; P_22A11_, 17,172 ± 53; P_32D8_, 25,405 ± 144; P_21B8_, 27,724 ± 70; P_33G9_, 36,462 ± 295; P_22D2_, 38,779 ± 180; P_33C6_, 42,278 ± 322; P_29F12_, 43,579 ± 148; P_32B7_, 43,985 ± 8; P_28B12_, 45,329 ± 233; and P_31H1_, 46,454 ± 266.

### Development and validation of a synthetic promoter-based CRISPRi system

**Fig 3:**
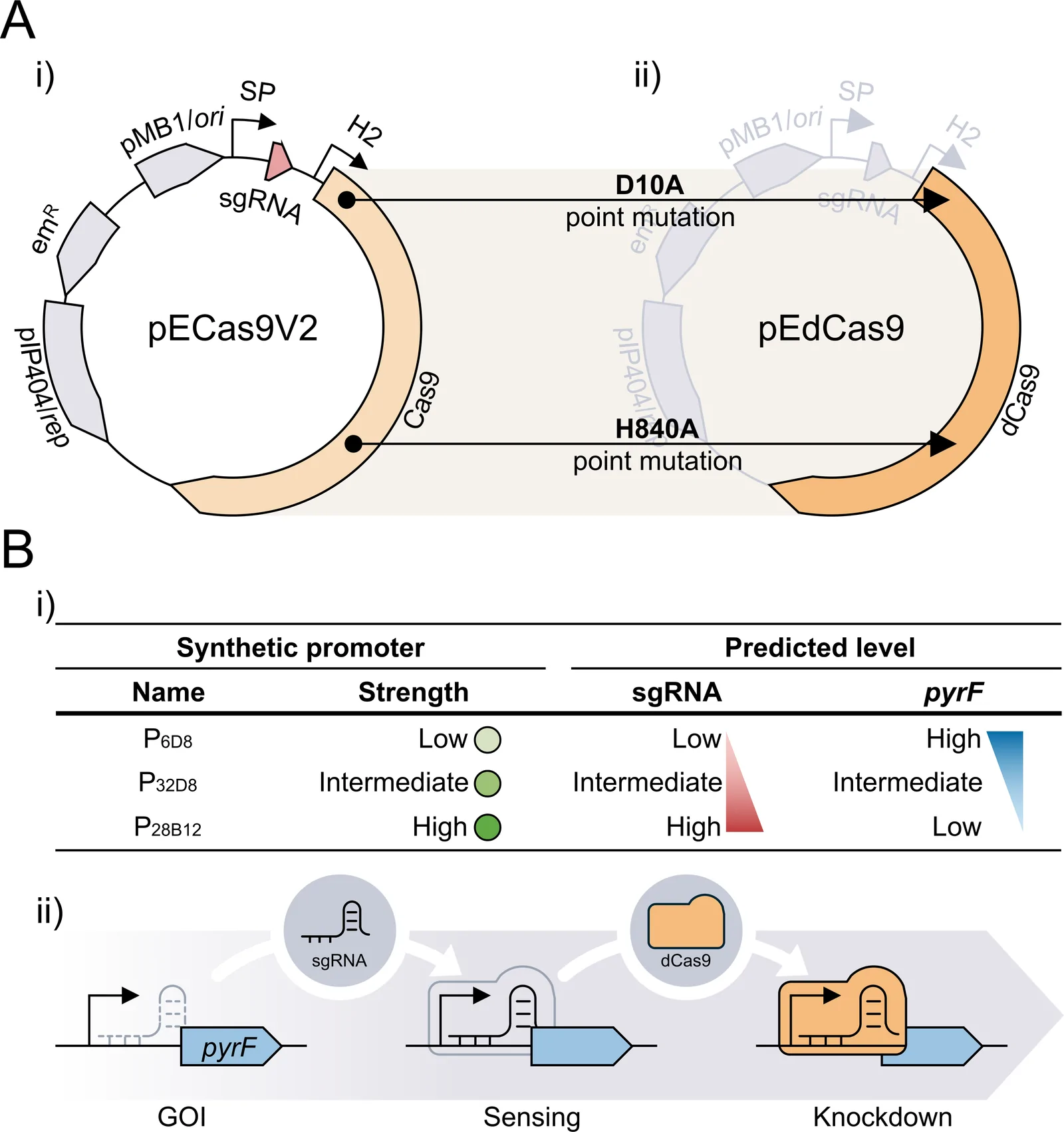
Construction and validation of the dCas9-based knockdown system integrating the synthetic promoter library. (**A**) The dCas9-carrying plasmid, pEdCas9, was generated by point mutations at two active sites (D10A and H840A) in Cas9 from pECas9V2. (**B**) The synthetic promoter library was used to control the strength of sgRNA transcription for fine-tuned *pyrF* expression. Three synthetic promoters from different groups (low, intermediate, and high) were selected to evaluate their impact on *pyrF* knockdown in *E. callanderi* KIST612. SP, synthetic promoter; GOI, gene of interest.

**Fig 4:**
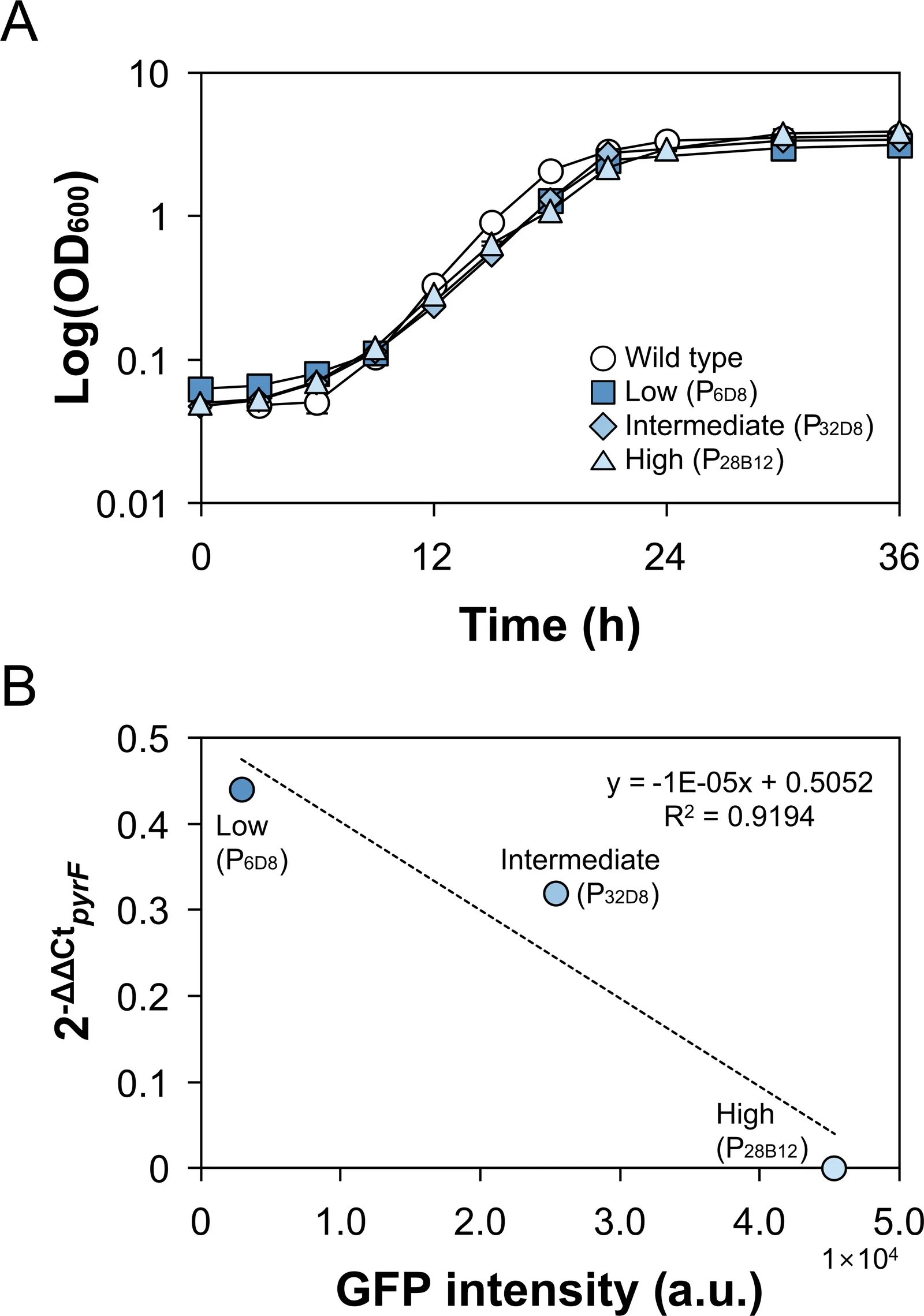
The effect of synthetic promoter strengths (low: P_6D8_; intermediate: P_32D8_; high: P_28B12_) on bacterial growth and *pyrF* knockdown efficiency. (**A**) *E. callanderi* wild-type and three *pyrF*-knockdown mutants (harboring pEdCas9::P_6D8_-sg_pyrF, pEdCas9::P_32D8_-sg_pyrF, or pEdCas9::P_28B12_-sg_pyrF) showed no significant differences in growth. (**B**) The *pyrF* transcription level was inversely proportional to promoter strength, showing a high correlation (*R*^2^ = 0.92). The transcription level of each mutant was measured by RT-qPCR. The GFP intensity of the corresponding promoter was derived from Fig. 2. GFP, green fluorescent protein; a.u., arbitrary unit.

We investigated the transcription levels of single-guide RNA (sgRNA) and the knockdown efficiency of genes of interest (GOI) as a function of promoter strength in the CRISPR/dCas9 system. The amount of sgRNA is a critical determinant of repression efficiency because dCas9 specifically binds to sgRNA targeting the GOI. Here, the synthetic promoter library was employed for tunable sgRNA transcription, and its effect on GOI transcription and expression were systematically analyzed. To construct the dCas9 knockdown system, point mutations (D10A and H840A, identified as key catalytic residues) were introduced into *Streptococcus pneumoniae* Cas9 gene from pECas9V2 (49) (Fig. 3A). For sgRNA transcription, P_j23119_ was replaced with promoters P_6D8_, P_32D8_, and P_28B12_ representing low-, intermediate-, and high-strength promoter groups, respectively. The ELI_4156 gene (orotidine-5′-phosphate decarboxylase, *pyrF*) was selected as a knockdown target, as its knockdown does not affect bacterial growth under uracil-supplemented conditions. In this system, the transcription level of mRNA *pyrF* was predicted to vary according to the promoter used (Fig. 3B). As a result, *E. callanderi* wild-type and the three *pyrF*-knockdown mutants displayed similar growth characteristics (Fig. 4A). Notably, *pyrF* transcription levels were inversely proportional to promoter strength (*R*^2^ = 0.92), independent of growth (Fig. 4B). P_6D8_, P_32D8_, and P_28B12_ decreased *pyrF* transcription to 0.440-, 0.319-, and 0.001-fold of the wild-type level, respectively, indicating that high-strength promoters show greater knockdown efficiency, while low-strength promoters allow milder repression.

**Fig 5:**
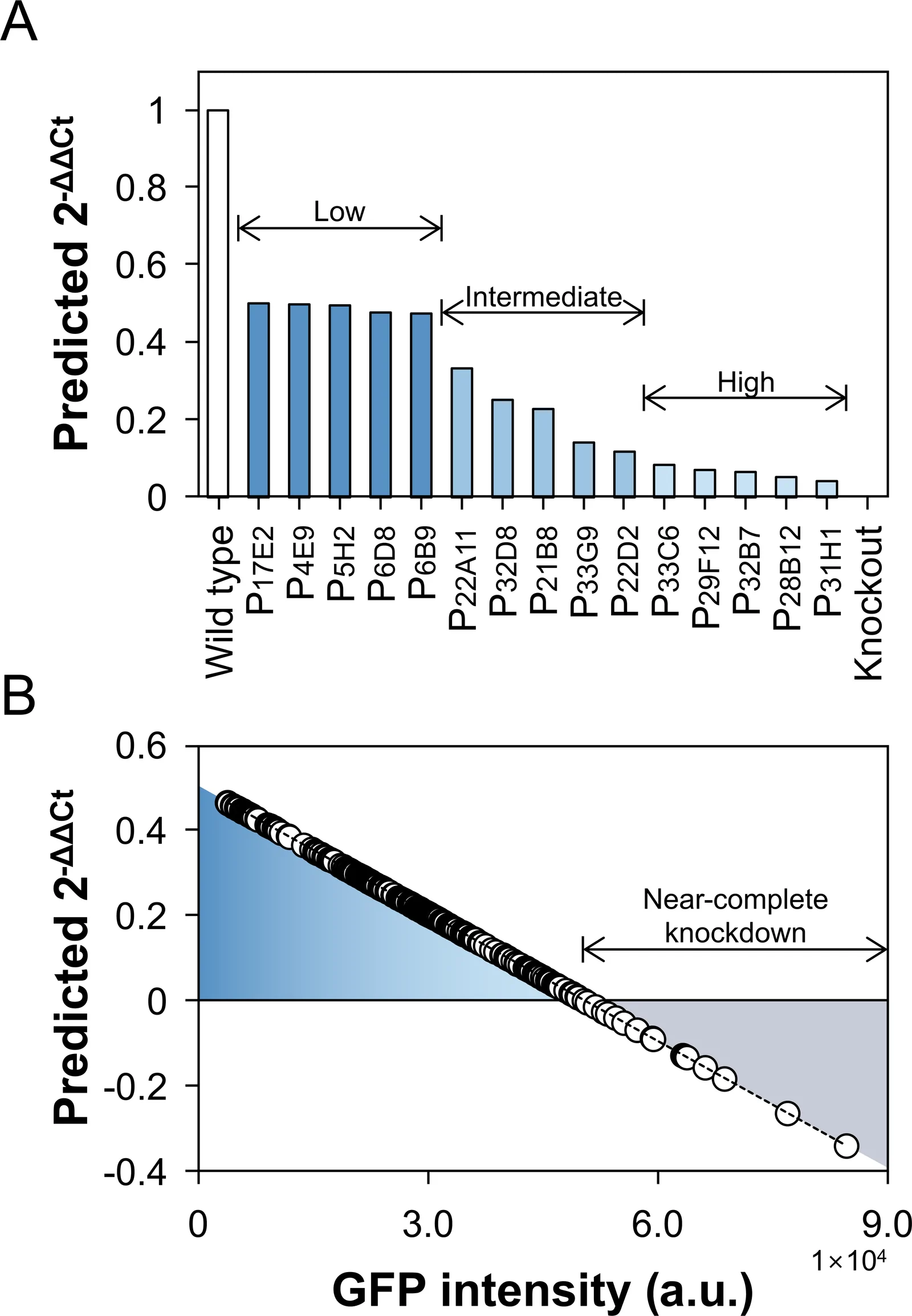
Prediction of knockdown efficiency using the dCas9-based knockdown system integrating the synthetic promoter library. The relative expression levels (2^-ΔΔCt^) of GOI for (**A**) 15 selected promoters and (**B**) the entire synthetic promoter library were calculated based on the high-correlation linear regression model established in Fig. 4. Note that 17 strong promoters in the high group yielded theoretical negative values in this prediction model. Subsequent experimental validation of these promoters confirmed negligible GOI expression, indicating a near-complete knockdown effect. GFP, green fluorescent protein; a.u., arbitrary unit.

Based on these results, knockdown levels for 15 synthetic promoters were predicted (Fig. 5A). The predicted transcriptional repression levels ranged from 0.041- to 0.500-fold relative to wild-type. Low- and high-strength promoters achieved approximately 50% (P_17E2_: 0.500, P_4E9_: 0.500, P_5H2_: 0.496, P_6D8_: 0.476, P_6B9_: 0.474) and 10% (P_33C6_: 0.082, P_29F12_: 0.069, P_P32B7_: 0.065, P_28B12_: 0.052, P_31H1_: 0.041) repression levels, respectively. Intermediate-strength promoters displayed a broader spectrum of repression (P_22A11_: 0.333, P_32D8_: 0.251, P_21B8_: 0.228, P_33G9_: 0.141, P_22D2_: 0.117), demonstrating their suitability for diverse gene repression requirements. Interestingly, a “near-complete knockdown” was predicted for promoters among the top 17 in strength, corresponding to GFP intensities exceeding 50,696 a.u. (Fig. 5B). We defined these as “near-complete repression promoters,” capable of achieving almost total transcription silencing. These promoters offer an alternative to conventional gene knockout methods, significantly reducing experimental complexity and time, especially in multi-gene knockout studies. Traditional knockout strategies are labor-intensive, experimentally demanding, and limited in performing simultaneous knockouts. In contrast, the near-complete knockdown system enables concurrent repression of multiple genes using knockdown vectors, facilitating rapid and efficient phenotypic analyses.

We next applied the established tunable knockdown system to *ldh* (ELI_1953), a gene involved in the metabolism of lactate, which is one of the major products under heterotrophic conditions. To achieve efficient *ldh* repression, we selected three representative promoters from different strength groups: P_22A11_ (high-strength, near-complete repression), P_32D8_ (intermediate-strength), and P_6D8_ (low-strength). The P_22A11_- and P_6D8_-based knockdowns were expected to show phenotypes similar to those of the *ldh* knockout and the control strain with the empty vector (pEdCas9), respectively. The P_6D8_-based mutant exhibited growth comparable to that of the wild-type, whereas growth inhibition was observed in the other mutants (Fig. S1A). As expected, the *ldh* mRNA level in each mutant was repressed in a promoter strength-dependent manner, consistent with the result of *pyrF* knockdown (Fig. S1B). Consequently, lactate production decreased in the three mutants, reflecting promoter strength (control: 0.60 ± 0.03; P_32D8_: 0.17 ± 0.02; P_6D8_: 0.30 ± 0.02 mmol/h/gCDW) (Fig. S1C). Notably, the P_22A11_-based mutant did not produce lactate, demonstrating the potential of “near-complete repression promoters” as an alternative to gene knockout. Consistent with observations in *pyrF* knockdown, we confirmed that sufficiently high promoter strength enables the dCas9-mediated “near-complete knockdown,” achieving effects comparable to gene deletion. In the high promoter-based *ldh* knockdown mutant, lactate production decreased by 100% relative to the control, providing the first phenotypic evidence of near-complete knockdown. Collectively, these results establish a versatile transcriptional regulation system optimized for KIST612, the model acetogen, enabled by the synthetic promoter library and CRISPR/dCas9-based knockdown approach developed in this study.

## DISCUSSION

This study established a synthetic promoter library and CRISPRi system for KIST612 and demonstrated its utility for precise synthetic promoter-CRISPRi-mediated target gene regulation. Motif analysis of 3,109 putative native promoters identified semi-conserved −35 and −10 elements, which guided the design of the synthetic promoter library. The library produced GFP intensities ranging from 3,864 to 84,568 a.u., spanning a dynamic range exceeding 20-fold (50). Among 15 representative promoters selected for detailed characterization, transcriptional strengths ranged from 544 ± 13 to 46,454 ± 266 a.u., corresponding to a 3.8- to 325-fold increase relative to the negative control. Compared to the limited genetic tools previously available for acetogens, these results confirm that promoter engineering enables the generation of predictable and finely tunable regulatory elements in acetogens (4, 40, 44, 48).

The library was first validated by targeting *pyrF* (40). Promoter-dependent transcription of sgRNA yielded graded repression of *pyrF*, from 0.440-fold (P_6D8_) to nearly complete silencing at 0.001-fold (P_28B12_) relative to wild-type levels. Regression analysis showed a strong correlation between promoter strength and knockdown efficiency (*R*² = 0.92). Importantly, repression did not affect growth under uracil-supplemented conditions, confirming gene-specific effects. These results establish a quantitative framework in which promoter strength directly determines CRISPRi efficacy, providing a tunable alternative to conventional gene knockout approaches. Overall, this study systematically quantified knockdown efficiency in the dCas9 system by precisely modulating sgRNA transcription through synthetic promoter strength (22, 49, 51). The developed system represents an efficient tool for functional genomics and phenotypic studies. Using the developed knockdown system, we generated three *ldh* mutants that exhibited distinct lactate production, which was strongly correlated with promoter strength. The P_6D8_-based mutant showed mild repression of *ldh*. In contrast, the P_22A11_- and P_32D8_-based mutants displayed dramatic changes in lactate profiles. Notably, the P_22A11_ completely inhibited lactate production, providing the first phenotypic evidence of a “near-complete knockdown” that could serve as an alternative to gene knockout.

This study provides a versatile and robust platform for functional genomics and phenomics, where precise and flexible control of gene expression is required. By enabling gene knockdown at diverse intensities, this system facilitates in-depth investigation of gene functions and regulatory networks. Specifically, the technical ease of modulating sgRNA expression through synthetic promoters—rather than modifying the large dCas9 protein—expedites the cloning process and facilitates the implementation of multiplex gene knockdown. Moreover, the discovery of near-complete knockdown-capable synthetic promoters could not only simplify single- and multiple-gene repression procedures but also be used for biotechnological applications, such as pathway optimization and microbial cell factory design. It would be a strong foundation for future advancements in microbial gene regulation, functional genomics, and synthetic biology, enabling precise and robust control of gene expression in various contexts.

## MATERIALS AND METHODS

### Strain cultivation

*E. coli* DH5α was cultivated at 37°C in LB medium. Chloramphenicol (25 μg/mL) or erythromycin (300 μg/mL) was used for selection of *E. coli* transformants. KIST612 was anaerobically cultivated at 37°C in 2× YTG. Erythromycin (120 μg/mL) was used for selection of KIST612 transformants. Cell growth was determined by measuring the optical density at OD_600_ using V-730 (JASCO, Japan). Gas compounds were measured by using a gas chromatography equipped with a thermal conductivity detector. Lactate was measured by using a gas chromatography equipped with a flame ionization detector, and acetate and butyrate were measured by using a high-performance liquid chromatography equipped with a refractive index detector.

### Bioinformatic analysis

The whole-genome sequence of *E. callanderi* KIST612 (CP002273) was obtained from the National Center for Biotechnology Information (25, 26, 52). RNA-sequencing data were previously performed by these authors (29, 40). Transcription units were predicted using Kyoto Encyclopedia of Genes and Genomes, OperonDB, and ODB4 (53–55). The promoter motif in the sequences was predicted using STREME of MEME Suite. The 5′ UTR (150 bp) from the (first) start codon of each transcription unit was used for promoter motif analysis.

### Plasmid construction

The pECas9V2 plasmid, a CRISPR/Cas9 expression vector specifically designed for *E. callanderi* KIST612, was used as the backbone for plasmid construction. To generate the dCas9 vector pEdCas9, point mutations (D10A and H840A) were introduced into the Cas9-coding sequence of pECas9V2 (22, 29, 51) (Table 3). The expression level of dCas9 was regulated by the inserted synthetic promoter. For the construction of sgRNA modules, DNA fragments were amplified using the primer sets xx-pyrFKD/sgTer_PvuI or xx-ldhKD/sgTer_PvuI (40) (Table S1). The amplified fragments were subsequently digested with SpeI and PvuI and ligated into the linearized pEdCas9 vector to complete the construction.

**TABLE 3:** Used plasmids in this study

Name	Relevant characteristics	Ref.
pECPH2::*uidA*	pMB1 *ori*, pIP404 *ori*, pIP404 rep, *em^R^*, H2, *uidA*	
pESP1-GFP	pMB1 *ori*, pIP404 *ori*, pIP404 rep, *em^R^*, synthetic promoter, GFP	This study
pECas9V2	pMB1 *ori*, pIP404 *ori*, pIP404 rep, *em^R^*, H2, Cas9	
pEdCas9	pMB1 *ori*, pIP404 *ori*, pIP404 rep, *em^R^*, H2, dCas9	This study
pEdCas9::P_6D8_-sg_pyrF	pMB1 *ori*, pIP404 *ori*, pIP404 rep, *em^R^*, H2, dCas9, P_6D8_, sgRNA	This study
pEdCas9::P_28B12_-sg_pyrF	pMB1 *ori*, pIP404 *ori*, pIP404 rep, *em^R^*, H2, dCas9, P_28B12_, sgRNA	This study
pEdCas9::P_32D8_-sg_pyrF	pMB1 *ori*, pIP404 *ori*, pIP404 rep, *em^R^*, H2, dCas9, P_32D8_, sgRNA	This study
pEdCas9::P_22A11_-sg_ldh	pMB1 *ori*, pIP404 *ori*, pIP404 rep, *em^R^*, H2, dCas9, P_22A11_, sgRNA	This study
pEdCas9::P_32D8_-sg_ldh	pMB1 *ori*, pIP404 *ori*, pIP404 rep, *em^R^*, H2, dCas9, P_32D8_, sgRNA	This study
pEdCas9::P_6D8_-sg_ldh	pMB1 *ori*, pIP404 *ori*, pIP404 rep, *em^R^*, H2, dCas9, P_6D8_, sgRNA	This study

### Synthetic promoter library construction

Plasmid backbone containing the synthetic promoter sequence (5′-ATCATTGACAWNNNNNNNNNNNNRTGNTATAATAAWADHA-3′) was obtained from pECPH2::*uidA* using K281 and K282 (40) (Table 3; Table S1). The GFP sequence (BBa_E0040) was amplified using K279 and K280. The amplified random DNA fragment and GFP backbone were assembled to construct pESP1-GFP using Gibson Assembly Master Mix (New England Biolabs, USA) according to the manufacturer’s instructions. The assembled plasmids were then introduced into *E. coli* DH5α via the standard heat-shock transformation method and plated onto agar plates to obtain single colonies.

### Reporter gene assay

To screen the synthetic promoter library, a total of 3,290 individual colonies were picked from the agar plates and separately inoculated into 96 deep-well plates containing 1 mL of LB medium. Following individual overnight cultivation at 37°C, the cells were immediately subjected to fluorescence measurement. GFP intensity and optical density (OD_600_) were quantified using FLx800 Microplate Reader (BioTek, USA) at 485/528 nm (bandwidth 20 nm) and Epoch (BioTek, USA), respectively. To account for variations in cell density, the fluorescence intensity of each sample was normalized by its corresponding OD_600_ value. For further validation, 15 selected promoters exhibiting high activity were re-evaluated using a FluoroMate FS-2 Spectrometer (SCINCO, Republic of Korea) at 488/510 nm (50, 56).

### Reverse transcription quantitative polymerase chain reaction (RT-qPCR)

Cells were grown to the mid-exponential phase in 2× YTG and were harvested at 10,000 × *g* for 20 min at 4°C. Total RNA was extracted using Ribospin II (GeneAll, Republic of Korea) and was quantified by measuring the A260/A280 ratio. Complementary DNA was synthesized from the extracted RNA using TOPscript cDNA Synthesis Kit (Enzynomics, Republic of Korea). RT-qPCR was performed using AccuPower GreenStar qPCR Premix (Bioneer, Republic of Korea) with primer sets RT_2048F/R, RT_4156F/R, or RT-sgpyrF/R (Table S1). The relative expression level of each transcript was calculated by the 2^-ΔΔCt^ method (57).

## SUPPLEMENTAL MATERIAL

Supplemental material spectrum.03779-25-s0001.pdf Table S1; Fig. S1.

ASM does not own the copyrights to Supplemental Material that may be linked to, or accessed through, an article. The authors have granted ASM a non-exclusive, world-wide license to publish the Supplemental Material files. Please contact the corresponding author directly for reuse.
