# Supplementary material for: A CRISPR interference system for tunable gene expression integrated with a promoter library for Eubacterium callanderi KIST612, an acetogen of functional diversity and versatility
Source: Microbiol Spectr. 2026 May 18;14(7):e03779-25. doi: 10.1128/spectrum.03779-25 (PMC13340025; doi:10.1128/spectrum.03779-25)
Supplement: Supplemental material — Table S1; Fig. S1. [file spectrum.03779-25-s0001.pdf]

Supporting Information

A CRISPR interference system for tunable gene expression integrated with a promoter library for *Eubacterium callanderi* KIST612, an acetogen of functional diversity and versatility

Byeongchan Kang[a,b], Ji-Yeon Kim[a,b], Soyoung Oh[a,b], Jina Kweon[a], Hongseo Park[a], Minji Kim[a], In-Geol Choi[c], and In Seop Chang[a,b]#

[a]Department of Environment and Energy Engineering, Gwangju Institute of Science and Technology, 123 Cheomdan-gwagiro, Buk-gu, Gwangju, 61005, Republic of Korea

[b]Research Center for Innovative Energy and Carbon Optimized Synthesis for Chemicals (inn-ECOSysChem), Gwangju Institute of Science and Technology, 123 Cheomdan-gwagiro, Buk-gu, Gwangju 61005, Republic of Korea

[c]Department of Biotechnology, College of Life Sciences and Biotechnology, Korea University, 145 Anam-ro, Seongbuk-gu, Seoul 02841, Republic of Korea

Running Head: CRISPR with synthetic promoter for KIST612

#Address correspondence to In Seop Chang, ischang@gist.ac.kr.

Byeongchan Kang and Ji-Yeon Kim contributed equally to this work.

Table S1. Used oligonucleotides in the study.

Name	Sequence (5’→3’)
K281	CAA ATA ATA AAT CAA CAA CTC TCC TGG CTA GCT AGC TAG CAG AAG TTG CAG AAT TAA GAA GAC AAC A
K282	CCT ATA GTC CAA ATT DHT WTT ATT ATA NCA YNN NNN NNN NNN NWT GTC AAT GAT ACT GGC CGT CGT TTT ACA GAC TAG TCA GAA TCA GGG GAT AAC GCA G
K279	AAT TTG GAC TAT AGG AGG TCT TTA TTA TGC GTA AAG GAG AAG AAC TTT TCA CTG G
K280	CCA GGA GAG TTG TTG ATT TAT TAT TTG TAT AGT TCA TCC ATG CCA TGT GTA ATC C
W5-pyrFKD	TTA CTA GTA TCA TTG ACA TAA ACC GTT AAC AAT GGT ATA ATA AAA TAA AGT ACC TGA AGG ATA AGG ACG TTT TAG AGC TAG AAA TAG C
H3-pyrFKD	TTA CTA GTA TCA TTG ACA ATC AAT TTC AGC GGT GAT ATA ATA AAA ACA AGT ACC TGA AGG ATA AGG ACG TTT TAG AGC TAG AAA TAG C
M3-pyrFKD	TTA CTA GTA TCA TTG ACA ACT ACC CGG CCT AAT GGT ATA ATA ATA ATA AGT ACC TGA AGG ATA AGG ACG TTT TAG AGC TAG AAA TAG C
sgTer_PvuI	TCG ATC GGC ACC GAC TCG GTG CCA CTT TTT CAA GTT GAT AAC GGA CTA GCC TTA TTT TAA CTT GCT ATT TCT AGC TCT AAA AC
RT_2048F	CTG GAA GAA ATT GAG ATT GAG GAG G
RT_2048R	CAA ATT TTT CTA CTG CCT TGA TCA GAC
RT_4156F	TTC TTC CGG GAA GGT CAG TC
RT_4156R	CCA GTA TGC CAA AGG CCA TTT T
RT_sgpyrF	AGT ACC TGA AGG ATA AGG ACG TTT TAG AGC
RT_sgpyrR	GTG CCA CTT TTT CAA GTT GAT AAC GGA CTA GCC TTA T

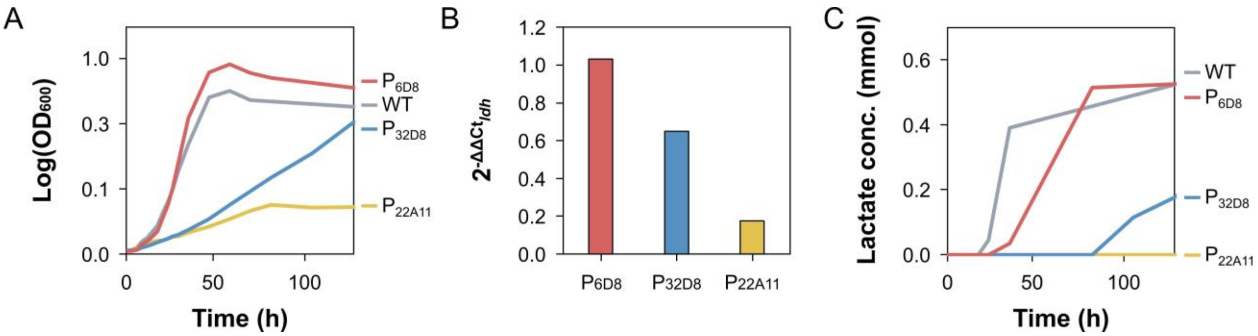

Figure S1. Lactate production properties by knockdown of three *ldh* mutants (P_{22A11}, P_{32D8}, and P_{6D8}). (A) Cellular growth of each mutant was determined by measuring OD_{600}. (B) The transcription level of each mutant was measured by RT-qPCR. (C) Lactate profile of each mutant was measured using HPLC.
